# Identification of Iron Homeostasis Genes Dysregulation Potentially Involved in Retinopathy of Prematurity Pathogenicity by Microarray Analysis

**DOI:** 10.1155/2015/584854

**Published:** 2015-10-18

**Authors:** Xian-qiong Luo, Chun-yi Zhang, Jia-wen Zhang, Jing-bo Jiang, Ai-hua Yin, Li Guo, Chuan Nie, Xu-zai Lu, Hua Deng, Liang Zhang

**Affiliations:** ^1^Department of Neonatology, Guangdong Women and Children's Hospital, Guangzhou 511400, China; ^2^Jinan University, Guangzhou 511000, China; ^3^Medical Genetics Center, Guangdong Women and Children's Hospital, Guangzhou 511400, China; ^4^Translational Medicine Center, Guangdong Women and Children's Hospital, Guangzhou 511400, China

## Abstract

Retinopathy of prematurity (ROP) is a serious disease of preterm neonates and there are limited systematic studies of the molecular mechanisms underlying ROP. Therefore, here we performed global gene expression profiling in human fetal retinal microvascular endothelial cells (RMECs) under hypoxic conditions *in vitro*. Aborted fetuses were enrolled and primary RMECs were isolated from eyeballs. Cultivated cells were treated with CoCl_2_ to induce hypoxia. The dual-color microarray approach was adopted to compare gene expression profiling between treated RMECs and the paired untreated control. The one-class algorithm in significance analysis of microarray (SAM) software was used to screen the differentially expressed genes (DEGs) and quantitative RT-PCR (qRT-PCR) was conducted to validate the results. Gene Ontology was employed for functional enrichment analysis. There were 326 DEGs between the hypoxia-induced group and untreated group. Of these genes, 198 were upregulated in hypoxic RMECs, while the other 128 hits were downregulated. In particular, genes in the iron ion homeostasis pathway were highly enriched under hypoxic conditions. Our study indicates that dysregulation of genes involved in iron homeostasis mediating oxidative damage may be responsible for the mechanisms underlying ROP. The “oxygen plus iron” hypothesis may improve our understanding of ROP pathogenesis.

## 1. Introduction

Retinopathy of prematurity (ROP) is a serious disease of preterm neonates and remains a leading causes of irreversible vision impairment or even blindness among children if untreated. This retinopathy is characterized by abnormal neovascularization during retinal development, subsequently leading to ROP in infants due to retinal vasospasm, ischemia, hypoxia, or hyperoxia. Two main mechanisms have been proposed to underlie the pathogenicity of ROP. First, cytokines such as vascular endothelial growth factor (VEGF), hypoxia inducible factor (HIF), and insulin-like growth factor-1 (IGF-1) were reported to induce ROP in murine models. Alternatively, excessive release of oxygen free radicals such as superoxide dismutase (SOD) and nitric oxide (NO) can impair retinal vasculature and induce retinal ischemia, particularly in preterm neonates and low-birth-weight (LBW) infants who are sensitive to oxidative stress because of deficient antioxidative protection [1]. Short gestational age, LBW, duration of oxygen therapy, and mechanical ventilation are considered risk factors for ROP progress. In addition to these environmental factors, there is also clinical and experimental evidence for a strong genetic basis for susceptibility to ROP, including sex, maternal ethnicity, and ocular pigmentation [2]. For example, VEGF, IGF-1, FZD4, and LRP5 gene polymorphisms were reported to be important genetic predictors associated with treatment and management of this disease. Despite these recent advances in the etiology and pathophysiology of ROP, there are limited systematic studies of the molecular mechanisms underlying ROP.

Microarray is a high-throughput technology for simultaneously examining expression of thousands of genes in a single experiment. Using this robust and effective tool, some murine models under hypoxic conditions to resemble human ROP were used to explore the underlying mechanisms. Recently, Pietrzyk and colleagues conducted genome-wide transcriptional profiling of peripheral blood in the first month of life of babies born with or without ROP. Pathway enrichment analysis indicated that downregulated inflammatory response genes were preferentially associated with ROP. Of note, the majority of genes differentially expressed exhibited only a 1.0–1.5-fold-change [3]. Using blood leukocytes often leads to high background signals in microarray and only a few genes with fold-change >2 were obtained in this study.

Because of ethical limitations, collection of human ROP subjects and controls is very difficult. It is well established that RMECs dysfunction is intimately implicated in the pathogenesis of several retinal vasoproliferative lesions, particularly in ROP, and that hypoxia is a key mediator in the development of ROP [4]. Thus, in the present study, we treated cultured primary human fetal RMECs with CoCl_2_, a commonly used chemical hypoxia-mimetic agent, and performed global gene profiling [5]. Our data suggest that hypoxic RMECs exhibit dysregulation of iron homeostasis, which may be an important mechanism of ROP pathogenicity.

## 2. Materials and Methods

### 2.1. Patients and Cell Culture

Eyeballs were taken from aborted foetuses with a mean gestational age of 20–28 weeks at the Guangdong Women and Children's Hospital between June and August 2012. Five subjects (three males and two females) with a mean weight of 610–2205 g were enrolled in the study. All parents gave written informed content, and the Institutional Ethics Board of Guangdong Women and Children's Hospital approved this study. Primary RMECs were isolated and maintained in DMEM media complemented with 10% FBS as described in our previous report [6]. Specific antibodies against von Willebrand factor (vWF) and CD31 were used to validate expression and localization of endothelial cell markers. Immunocytochemical staining and confocal immunofluorescence microscopy assay were performed to identify the origin of these cells. When RMECs reached 70–80% confluence, CoCl_2_ (Sigma, St. Louis, MO, USA) was added to the media at 100 *μ*M, 150 *μ*M, 200 *μ*M, 250 *μ*M, 300 *μ*M, or 350 *μ*M for 12 h, 24 h, 48 h, 72 h, or 96 h, respectively. MTT assay is described elsewhere and a modified procedure was used to assess cell viability. Cultured cells in 200 *μ*L media were seeded into the 96-well plates, and 100 *μ*L MTT (5 mg/mL) was added. After incubation at 37°C in 5% CO_2_, 100 *μ*L DMSO was pipetted to solubilise the formazan product at room temperature. The absorbency was measured using microplate reader (Bio-Rad). The growth curves were plotted according to MTT data and cells without CoCl_2_ treatment were used as controls.

### 2.2. Microarray Analysis

Total RNA extracted from cultured RMECs treated with 150 *μ*M CoCl_2_ for 72 h was reversely transcribed to cDNA and amplified using the MessageAmp II aRNA Amplification Kit (Life Technologies, Carlsbad, CA 92008, USA) according to the manufacturer's recommendations. Comparisons of gene expression profiling were performed between CoCl_2_-treated RMECs and the paired untreated control. The dual-color microarray approach was adopted in our study, as follows. After a series of enzyme reactions, the derivative cDNA of CoCl_2_-treated cells labeled with Cy5 and the cDNA of untreated cells labeled with Cy3 were combined and hybridized to the microarray. To assess and minimize the effects of dye bias on fluorescent hybridization signals, dye-swap assays were performed. Microarray hybridization was performed on Agilent SurePrint G3 Human GE 8 × 60 K Microarray Kit (Agilent Technologies, Santa Clara, CA, USA). The arrays were washed consecutively using Gene Expression Wash Buffer Kit (Agilent Technologies) after hybridization. Fluorescence images of the hybridized arrays were generated using the Agilent DNA Microarray Scanner, and the data of obtained images were extracted with Agilent Feature Extraction software.

### 2.3. Quantitative RT-PCR Verification

Quantitative RT-PCR (qRT-PCR) was used to validate the accuracy of the results obtained by microarray analysis. First-strand cDNA was synthesized from 0.5 *μ*g total RNA using Thermoscript RT (Invitrogen, Carlsbad, CA, USA) and oligo dT primers. PCR amplification was conducted with the LightCycler 1.5 (Roche Diagnostics, Tokyo, Japan) following the manufacturer's instructions. The experiments were repeated three times. Genes were randomly selected from the group of DEGs with a fold-change >2 in the microarray experiment.

### 2.4. Statistical Analysis

Genes with the signal intensity >800 (Cy5 or Cy3) were regarded as expressed genes. The fold-change of the Cy5 to Cy3 of each spot was calculated after normalization through Agilent Feature Extraction. For unsupervised hierarchical clustering, we applied average-linkage hierarchical clustering and TreeView to visualize the results. The one-class algorithm was used to screen the differentially expressed genes (DEGs) with a threshold set at fold-change >2.0 and *q*-value <0.05. Functional enrichment analysis based on Gene Ontology was used for classifying DEGs associated with biologically relevant functional categories and canonical pathway involvement.

## 3. Results

### 3.1. CoCl_2_-Induced Hypoxia in Primary RMECs

**Figure 1:**
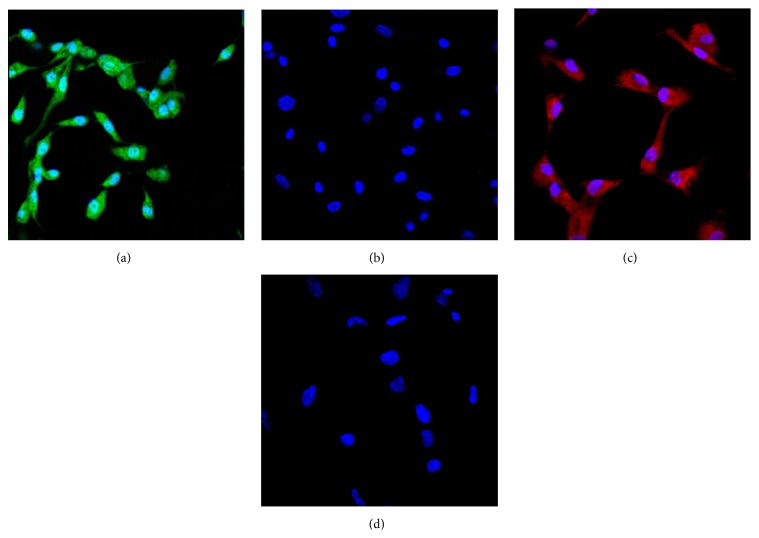
Confocal images of cultured human RMECs. (a) Immunofluorescent staining of vWF with sheep polyclonal antibody (Abcam). (b) Negative control of vWF identification. (c) Immunofluorescent staining of CD31 with mouse monoclonal antibody (Abcam). (d) Negative control of CD31 identification.

After mechanical isolation and enzymatic digestion, primary cells were released from retinal tissue fragments after 48 h seeding and then readily expanded and formed a cobble stone-like monolayer. To confirm that the cultured cells were originated from vascular endothelial cells, expression of vWF and CD31 was detected by confocal immunofluorescence microscopy. Almost all of the cells showed strong staining for both vWF and CD31 in the cytoplasm, indicating the high purity (95–97%) of vascular endothelial cells (Figure 1). The cells were maintained in culture for 5–7 passages without a significant loss in expression of these two markers.

**Figure 2:**
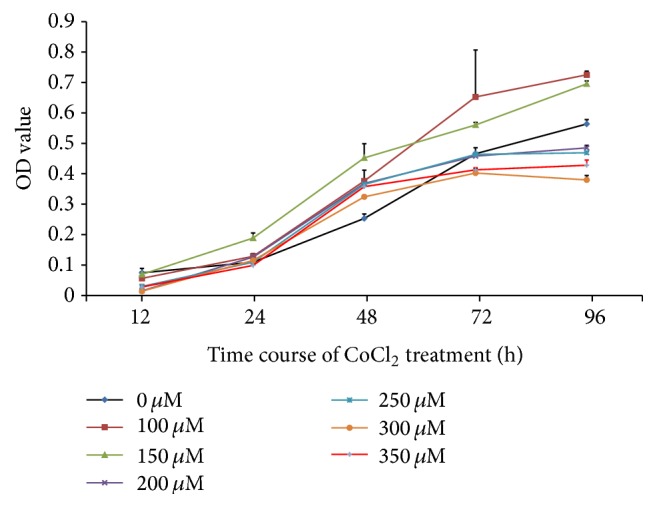
Growth curve of human RMECs treated with various concentration of CoCl_2_.

In our pilot experiment, RMECs exposed to different concentrations of CoCl_2_ exhibited distinct growth activities. As shown in Figure 2, growth promotion of 100 *μ*M and 150 *μ*M CoCl_2_-treated cells was observed during the time course (12–96 h). At a concentration of CoCl_2_ > 150 *μ*M, cell growth was suppressed after 72 h incubation. Therefore, we selected 150 *μ*M CoCl_2_ treatment for the hypoxia-induced study.

### 3.2. Comparison of Gene Expression Profiles between the Two Groups

**Figure 3:**
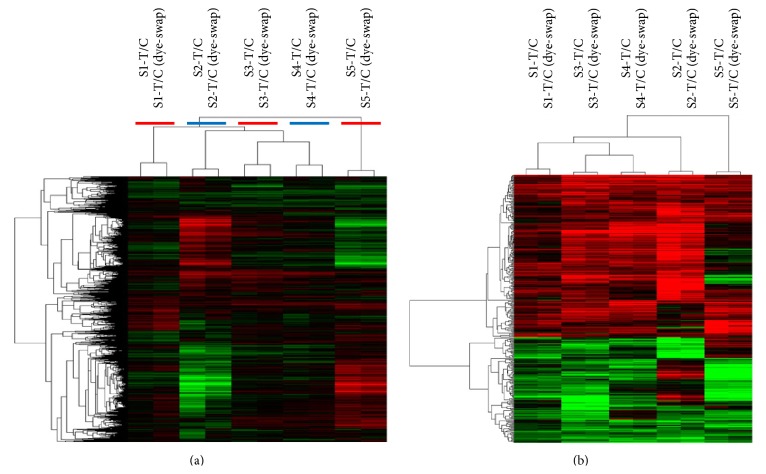
Heat map of gene expression pattern of five pairs of human RMEC subjects with dye-swap. The red color indicates upregulation and green color indicates downregulation. (a) Unsupervised clustering of 15,400 genes with a signal intensity >800 in matched RMEC samples. (b) A total of 326 (198 upregulated and 128 downregulated). DEGs in hypoxia-induced RMEC, compared with normoxia control (fold-change >2 and *q* < 0.05 with the SAM analysis).

A total of 15,400 genes with the signal intensity >800 were screened for unsupervised clustering. Dye-swap assays showed that the gene expression patterns of each pair of samples were highly similar, exhibiting the excellent technical reproducibility of our microarray experiments (Figure 3(a)). The microarray raw data were submitted to Gene Expression Omnibus with an accession number of GSE59462. There were 326 DEGs between the hypoxia-induced group and control group using SAM software (one class) that exhibit the combined criteria of a *P* value <0.05 and fold-change >2 (Figure 3(b)). Of these genes, 198 were upregulated in the hypoxia-induced RMECs, while 128 were downregulated.

**Figure 4:**
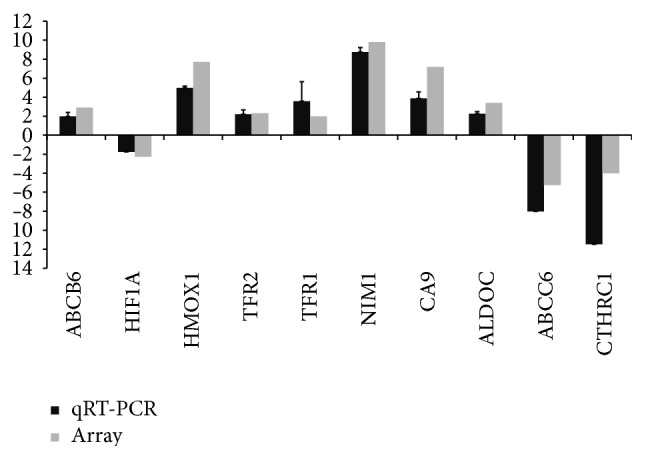
Comparison of gene expression changes from microarray and qRT-PCR.

Gene Ontology analysis showed highly enriched signaling pathways with altered gene expression in RMECs under hypoxic condition, the top six of which were glycolysis, carbohydrate metabolic process, iron ion homeostasis, response to hypoxia, type I interferon-mediated signaling pathway, and positive regulation of angiogenesis (Table 1). Notably, iron homeostasis pathway genes with altered expression included* HMOX1, TFR2, TFR1, FTHL17, FTMT, FTH1, FTL, ABCB6, HIF1A*, and* MFI2* (Table 2). Moreover, a total of ten altered expressed genes, including* ABCB6, HIF1A, HMOX1, TFR2, TFR1, NIM1, CA9, ALDOC, ABCC6, and CTHRC1*, were selected for qRT-PCR. qRT-PCR analysis was in good agreement with the data from the microarray in our experiments (Figure 4).

## 4. Discussion

In this study, global expression profiling was employed to compare the transcriptional pattern between hypoxia-induced RMECs and controls. To ensure the validity of this assay, we performed both biological replicates (five pairs of subjects) and technical replicates (dye-swap). A total of 326 genes from 15,400 expressed hits were defined as DEGs, with 198 upregulated and 128 downregulated. Furthermore, the highly consistent results between qRT-PCR and microarray assays demonstrated the reliability and reproducibility of our microarray platform (Figure 4). As for sample size, it depends on different types of research studies. Usually, hundreds or thousands of subjects would be favorable when identifying the genes involved in susceptibility to disorders, or finding gene classifier for disease subtypes. However, when imploring the molecular mechanism of disease at the expression level, the biological replicates were not required as much as the aforementioned two fields. In general, 3–5 biological independent replicates in this kind of studies are reasonable and acceptable and published articles already justify this issue. Therefore, we tried to collect 5 aborted foetuses to isolate human RMECs for* in vitro* assay. To the best of our knowledge, this is the first study for ROP using this kind of samples.

ROP is retinal vascular proliferative disease in newborns of prematurity. Cultured RMECs from the bovine and rat have been previously used for biological studies [7]. Primary RMECs from aborted fetuses would be suitable and ideal for* in vitro* ROP experiments. In the present study, we first evaluated whether CoCl_2_ administration could mimic hypoxia in RMECs. As expected, genes associated with responses to hypoxia according to Gene Ontology term exhibited significantly altered expression, including* ADM, ALDOC, ANG, HIF1A, ASCL2, MT3, ANGPTL4, PLAU, PLOD2, EGLN1, BNIP3, VEGFA, NDRG1, HMOX1, STC1, FNDC1*, and* CCNB1* (13/17 of these were upregulated). The other top enriched pathways also included glycolysis, carbohydrate metabolic process, and positive regulation of angiogenesis, indicating that the RMECs can adaptively respond to hypoxic stress. These adaptive responses are in good accordance with CoCl_2_-induced hypoxia.

Of note, we found that* HIF1A* mRNA was downregulated by microarray and qRT-PCR. Many reports have shown that* HIF1A* mRNA is elevated or remains unchanged under oxygen-deficient conditions. However, the effect is dependent on the concentration of CoCl_2_, duration of treatment, severity of hypoxia, and different cell types. Additionally,* HIF1A* mRNA is believed quite unstable and its expression occurs in a temporal-spatial manner. For example, in human lung microvascular endothelial cells and A549 cancer cells,* HIF1A* mRNA level dramatically diminished from 4–12 h of prolonged hypoxia when cells were exposed to CoCl_2_ (250 *μ*M), despite a marked induction of* HIF1A* protein expression over the same time course [8]; this was mainly related to a reduction in* HIF1A* mRNA stability and an increase in natural antisense* HIF1A*. Another study also described a decrease in* HIF1A* mRNA level at 8 h in Hep 3B cells subjected to 75 *μ*M CoCl_2_ [9]. In the present study, we treated human RMECs with 150 *μ*M CoCl_2_ for 72 h with a similar result. In addition, some classic* HIF1A* downstream targets have been reported, including* VEGFA, GLUT1, ALDOA, HMOX1*, and* LDHA*, which were markedly upregulated in our experiments. Collectively, these results strongly indicate that the induced* HIF1A* by CoCl_2_ treatment was able to activate transcription and mediated the downstream hypoxia signalling cascades in RMECs.

Since regulation of glycolysis and carbohydrate metabolism pathways is commonly reported under hypoxic conditions, we further examined the third-ranked iron ion homeostasis pathway in our study (Table 1). All targets with changed expression including* HMOX1, TFR2, TFR1, FTHL17, FTMT, FTH1, FTL, ABCB6, HIF1A,* and* MFI2* (8/10 upregulated except the last two genes) are shown in Table 2. Iron, the major trace element in living organisms, is a cofactor of many functionally important enzymes in normal biological processes and plays a role in DNA synthesis, oxygen transportation, energy metabolism, and detoxification, which require iron-containing proteins in mammals. However, free iron is particularly potent in inducing oxidative stress, as it promotes generation of reactive oxygen species (ROS) through the Fenton reaction, which damages impairs lipids, protein, DNA, and carbohydrates to impair cellular function and integrity. Cellular iron homeostasis is a tight and fine-tuned process involving a series of complex molecule interactions. These iron regulatory proteins include transferrin and transferrin receptor for iron import, ferroportin for export, and ferritin for storage. Indeed, iron and some molecules implicated in iron metabolism have been previously detected in the retina [10, 11].

Beginning in the 1980s, blood transfusion was recognized as an independent risk factor for the development of ROP in preterm infants, and recent publications also supported this association [12–15]. In general, there are several common explanations. First, transfusions increase oxygen delivery to the retina. This is due to the lower oxygen affinity of adult hemoglobin compared with fetal hemoglobin F [16]. Second, transfusions lead to iron overload. Packed red blood cells contain large amounts of iron and transfused cells have a shortened half-life. Nontransferrin bound iron in plasma were significantly increased in preterm infants after blood transfusion [17]. In addition, preterm infants have reduced protection against free iron because of their relatively low transferrin levels with concomitant a deficiency of iron binding capacity. Collectively, preterm infants are particularly prone to such free iron accumulation. Increased amount of free iron has the potential to promote the generation of highly reactive oxygen radicals capable of damaging the immature retina, which may contribute to the association of clinical transfusion with ROP. However, these theoretical considerations cannot explain some phenomena or findings, and the precise mechanism of ROP involved remains incompletely understood. Herein, our global transcriptomic data revealing the dysregulation of iron homeostasis pathway in fetal retinal cells may provide an insight into the pathogenesis of ROP.

HMOX1, heme oxygenase 1, is an essential enzyme in heme degradation and iron metabolism. In the present study, CoCl_2_ mimicked hypoxia and stabilized HIF1A, which can result in transcriptional activation of HMOX1 expression and subsequent facilitation of heme catabolism and oxygen homeostasis. Altered expression of HMOX1 is also implicated in iron recycling and iron homeostasis. Further, HMOX1 confers cytoprotection against different insults including hypoxia, oxidative stress, and iron overload. These effects occur as a result of breakdown of prooxidant heme to free iron, CO, and biliverdin, and the antioxidant activities of these byproducts of this reaction. Thus, these protective actions of HMOX1 are commonly considered to underlie the final outcome under any stress.

However, there is some debate over whether HMOX1 upregulation can solely be considered beneficial, as there is some evidence of adverse actions [18]. Indeed, it was previously reported that there is a threshold of HMOX1 overexpression that determines its beneficial or deleterious effects under given conditions [19]. Low overexpression of HMOX1, for example, 1.8-fold, was related to cytoprotection against heme-mediated damage and oxygen toxicity, whereas a 3.6-fold increase in HMOX1 expression resulted in a loss of protection function in fibroblasts [20]. Importantly, higher upregulation of HMOX1 was also associated with the accumulation of reactive iron released in the decomposition of heme, resulting in increased cytotoxicity in endothelial cells. In some circumstances such as endotoxin-induced shock, a significant overexpression of HMOX1 can also induce mitochondrial dysfunction because of elevated levels of catalytically active intracellular iron [21]. Further, it was recently reported that enhanced cardiac oxidative activity and apoptosis were related to* HMOX1 *mRNA and protein upregulation in macrophages [22]. The retina is a highly oxygenated tissue in the body and is especially sensitive to oxidative damage. Further,* HMOX1* mRNA expression was reported to gradually increase in the rat retina from 6 days before birth until the day of birth [23]. Thus, the overexpression of* HMOX1* (8.5-fold increase) in hypoxic RMECs in the present study may contribute to ROP development via oxidative stress-mediated injury in the retina.

FTMT (ferritin mitochondria), FTL (ferritin, light polypeptide), FTH1 (ferritin, heavy polypeptide 1), and FTHL17 (ferritin, heavy polypeptide-like 17) belong to the ferritin family. Ferritins are highly conserved intracellular iron-storage proteins. Cytosolic ferritin consists of 24 subunits of the heavy (H) and light chains (L). The H subunit is responsible for ferrous iron oxidation via regulation of ferroxidase activity, whereas the L subunit lacks this enzymatic feature. Various compositions of the ferritin subunit may have different effects on iron storage and release. A major function of ferritin is the sequestration of free iron in a soluble state to prevent generation of oxygen free radicals. FTMT, a newly discovered ferritin heavy chain-like protein, is located in the mitochondria. FTMT plays an important role in intracellular iron homeostasis as mitochondria are critical for cellular iron metabolism; for example, heme is synthesized in mitochondria. FTMT was also suggested to protect mitochondria from oxidative injury, as mitochondria exhibit high oxygen consumption and metabolic activity. However, overexpression of FTMT was found to contribute to translocation of iron from the cytosol to mitochondria [24]. Moreover, elevated expression of MTFT exerted an oxidative toxic effect on cells via mitochondrial iron accumulation and cytosolic iron uptake increase [25].

Interestingly, upregulation of ferritin mRNA, HMOX1, and TFR was found to be associated with elevated iron acquisition in FTMT-induced cells. Induction of HMOX1 and consequent iron release was also correlated with elevated ferritin level. Of note, oxidative stress can produce different effects on ferritin expression at the mRNA and protein levels; for example, oxidants can activate ferritin transcription but inhibit its translation by diverse modulation mechanisms. Our microarray results showed elevated mRNA expression of several ferritin members including FTL, FTH1, and FTHL17, in good agreement with previous reports. Nevertheless, upregulation of ferritin protein did not infer increased cytoprotection in cells overexpressing HMOX1. Indeed, an increase in oxidative damage was found, indicating that ferritin iron may be involved in ROS production when iron is released from ferritin [26, 27]. In some situations, ferritin could be a source of iron for oxidative injury, although the detailed mechanisms remain unclear. In addition, inconsistent effects of FTH1 expression on cell function have been reported. For example, induced FTH1 was shown to enhance resistance to oxidative injury, but it leads to iron overload and subsequent increase of ROS [28]. These discrepancies may relate to the different cell types used, the extent of FTH1 induction, and the various conditions used.

TFR1/TFRC, transferrin receptor, facilitates intracellular iron import from transferring. Posttranscriptional control via iron-responsive elements/iron regulatory protein (IRE/IRP) system plays a critical role in the regulation of TFR expression, which has been described in Tracey Rouault's papers [29, 30] and other literatures [31, 32]. Expression of the TFR1 is usually inversely regulated by intracellular iron levels. When intracellular iron is plentiful, IRP1 and IRP2 are not able to bind to the IRE in the 3′-untranslated region of* TFR1* mRNA and consequently lead to a rapid degradation of it. However, iron is not the only species that modulates TFR1 expression. In fact, transcriptional mechanism is also involved in TFR1 expression.* TFR1* transcriptional expression is upregulated by the HIF-1A via binding to hypoxia-responsive elements (HRE) of its mRNA. As a global regulator response to hypoxia, HIF-1A affects iron/erythropoiesis pathway [33, 34]. For example, exposure of Hep3B, K562, and HeLa cells to hypoxia or being treated with CoCl_2_ resulted in a 2- to 3-fold increase in* TFR* mRNA expression [35, 36]. In addition, increased expression of TFR was identified in CNS endothelial cell (EC) within 24 h of exposure to hypoxia, reflecting the tight molecular links between oxygen homeostasis and iron metabolism [37]. Thus, the coordination of transcriptional and posttranscriptional mechanisms decides the final TFR1 expression levels under specific conditions, which is consistent with the “oxygen plus iron hypothesis.”

TRF2, a homologue of TFR1, is not the major importer of iron into cells. It primarily modulates hepcidin levels, which is a key hormone that is involved in the control of iron homeostasis [38, 39]. Usually TFR2 is expressed in the liver, but it was also found in the mouse retina by RT-PCR and* in situ* hybridization [40]. Oxidative stress plays a key role in the pathogenesis and development of AMD. Increased iron acquisition through the transferrin-TFR pathway is involved in cell cytotoxicity caused by oxidative damage [41]. Upregulation of TFR combined with FTMT, HMOX1, and ferritin in RMECs may also lead to cellular oxidative toxicity in the retina and contribute to the etiology of ROP. Collectively, these findings suggest that the coordination and integration of a cluster of genes with altered expression involved in iron metabolism determine the final status of cellular iron.

Numerous lines of evidence have proven that HIF1A acts as a central regulator response to hypoxia and mediates transcriptional activation of many target genes in most cell types. Further, oxidative stress is closely associated with ROP [42]. The results of the present study may provide an insight into the link between these events. CoCl_2_ imitates hypoxia-induced HIF1A, which subsequently activated transcription of target genes implicated in iron homeostasis. Deregulation of iron metabolism can lead to ROS generation. As a consequence, oxidative damage contributes to the pathogenicity and development of ROP. The “oxygen plus iron” hypothesis may improve our understanding of the mechanisms underlying ROP.

In summary, we isolated and cultivated human retinal microvascular endothelial cells from aborted fetuses. Primary RMECs were treated with CoCl_2_ *in vitro* to mimic the pathogenicity of ROP. Global gene expression profiling revealed that the DEGs were highly enriched in the iron homeostasis pathway. Dysregulation of iron metabolism signaling may result in cellular iron accumulation and subsequent oxidative damage in retinal endothelial cells. Iron-chelating or antioxidant therapy may be beneficial for ROP management and prevention.

## Figures and Tables

**Table 1:** The predominant enriched pathways with DEGs by Gene Ontology analysis.

Go terms	Number of DEGs	*P* value	Enrichment factor	*Q* value
GO:0006096 (glycolysis)	10	1.27*E* − 09	14.3828799	4.03*E* − 07
GO:0005975 (carbohydrate metabolic process)	20	3.45*E* − 08	4.39476886	2.86*E* − 06
GO:0006879 (cellular iron ion homeostasis)	9	6.05*E* − 07	9.04066736	3.35*E* − 05
GO:0001666 (response to hypoxia)	13	2.95*E* − 06	4.86805166	0.00012243
GO:0060337 (type I interferon-mediated signaling pathway)	8	5.67*E* − 06	8.16576407	0.00018823
GO:0045766 (positive regulation of angiogenesis)	7	2.42*E* − 05	8.05441274	0.00061759

**Table 2:** List of genes with altered expression involved in iron ion homeostasis.

Gene symbol	Fold change	*Q* value (%)	Gene description
HMOX1	8.53	0	Heme oxygenase (decycling) 1
FTL	2.93	0	Ferritin, light polypeptide
FTMT	2.82	0	Ferritin mitochondrial
FTH1	2.33	0	Ferritin, heavy polypeptide 1
FTHL17	2.33	0	Ferritin, heavy polypeptide-like 17
ABCB6	2.88	0	ATP-binding cassette, subfamily B, member 6
TFR2	2.16	0	Transferrin receptor 2
TFR1	2.00	0	TFRC, transferrin receptor
MFI2	−2.04	0.17	Antigen p97 (melanoma associated)
HIF1A	−2.27	0	Hypoxia inducible factor 1, alpha subunit

## Abbreviations

ROP: Retinopathy of prematurity
RMECs: Retinal microvascular endothelial cells
HIF: Hypoxia inducible factor
DEGs: Differentially expressed genes
SAM: Significance analysis of microarray
qRT-PCR: Quantitative RT-PCR
ROS: Reactive oxygen species.
